# The relationship between nursing workload and quality of care in intensive care units

**DOI:** 10.62675/2965-2774.20250381

**Published:** 2025-04-16

**Authors:** Arnaud Bruyneel, Alberto Lucchini, Jérôme E. Dauvergne

**Affiliations:** 1 Université Libre de Bruxelles School of Public Health Hospital Management and Nursing Research Bruxelles Belgium Department of Health Economics, Hospital Management and Nursing Research, School of Public Health, Université Libre de Bruxelles - Bruxelles, Belgium.; 2 Fondazione IRCCS San Gerardo dei Tintori Department of Emergency and Intensive Care Monza Italy Department of Emergency and Intensive Care, Fondazione IRCCS San Gerardo dei Tintori - Monza, Italy.; 3 Nantes Université Laënnec Hospital Department of Anaesthesiology and Critical Care Nantes France Department of Anaesthesiology and Critical Care, Laënnec Hospital, Nantes Université - Nantes, France.

The World Health Organization (WHO) defines quality of care as the degree to which health services provided to individuals and populations increase the likelihood of the desired health outcomes. Quality care, particularly in critical care settings, must be effective, safe, and patient-centered. Indicators such as mortality, length of stay (LOS), and readmission are often used to measure quality.^(1)^

To ensure adequate and safe staffing levels in intensive care units (ICUs), international recommendations for nurse-to-patient (N:P) ratios have been established.^(2)^ However, applying these recommendations within a single country can be challenging because of variations in ICU definitions, ICU nurse autonomy levels, organizational structures, capacity strain, and admission triage processes.^(3)^

While the N:P ratio is a basic approach for estimating workload, it does not reflect the full complexity of ICU care. Additionally, since nursing staff constitutes a substantial portion of ICU costs - up to 60% - accurate assessment of appropriate nurse staffing levels is crucial.^(4)^ Tools such as the Nursing Activities Score (NAS) have been developed to provide a more accurate assessment of nursing workload. The NAS was introduced in 2003 when ICU technology was relatively primitive compared to today's sophisticated systems. According to two literature reviews, the NAS demonstrates the strongest content validity among workload measurement tools. Multi-moment recordings have shown that a comprehensive list of nursing activities accounts for 81% of nurses’ time.^(5,6)^ The NAS measures both direct and indirect care activities, as well as non-care-related tasks. Scoring can be recorded by shift or daily, with a range from 21 to 177%, indicating the proportion of nursing time required (100% = one nurse). Since its development, the NAS has been widely used internationally,^(7)^ although its intra- and inter-rater reliability suggest there is room for improvement. Updated guidelines for NAS implementation were released in 2015; however, there is a need to revise the NAS and its associated scoring system.^(7)^ This revision is necessary to better reflect the changing landscape of clinical practice and its effects on nursing workload and to further analyze evidence of validity from a contemporary perspective. Despite its limitations and the development of alternative methods, the NAS remains the most suitable instrument for evaluating workload.^(8)^

Studies on ICU workload have revealed a strong association between unfavorable N:P ratios and a decline in care quality, including higher rates of hospital-acquired infections, adverse events, readmissions, LOS, and ICU mortality.^(9)^ However, while it is easy data to collect, N:P ratios provide only aggregate data, overlooking key aspects such as nurse experience and workload complexity. For instance, an experienced team with a moderate workload is not equivalent to a less experienced team that handles a high workload at the same N:P ratio. A study in the Netherlands showed that an increase in NAS per nurse is associated with higher patient mortality and adverse events.^(10)^ Moreover, studies indicate that improving nursing staffing in ICUs is cost-effective for health insurers, as it decreases both LOS and readmission risk.^(11)^ Consequently, WHO is urging substantial investment in the nursing profession.^(12)^

The prevalence of missed care can largely explain this relationship between workload and patient outcomes. "Missed care" is an umbrella term encompassing several concepts (e.g., care left undone and unfinished care), and describes situations in which nurses are forced to delay or omit necessary nursing care.^(13)^ For instance, a nurse who does not have time to disinfect their hands could contribute to a care-related infection in one or more patients.

Several factors beyond workload influence the quality and organization of ICU nursing care. Higher levels of nurse education are correlated with improved patient outcomes.^(14)^ Specialized nurses also enhance the quality of care and patient satisfaction.^(15)^ A Brazilian study reported that increased ICU nurse autonomy positively affects patient outcomes.^(16)^ Autonomy encompasses more than task execution, enabling nurses to participate in decision-making, thus fostering a positive work environment and enhancing staff retention.^(17)^ Nurses’ clinical competencies are essential in managing workload and ensuring the delivery of high-quality care. A strong initial education, combined with regular continuing education, equips nurses to handle complex patient needs and adapt to advancements in healthcare practices. Ongoing training not only strengthens their expertise but also enhances their ability to organize work efficiently and mitigate risks associated with care errors. This approach is critical in maintaining patient safety and ensuring optimal outcomes, particularly in high-pressure healthcare environments.^(18)^ Additionally, experience and teamwork are essential for quality care delivery.^(19)^ Advanced nursing practice could also help to manage workload and improve patient outcomes. Nurses practicing in advanced roles can manage complex care needs more effectively, allowing for better task delegation and a more balanced workload. These skills can improve patient outcomes by providing specialized interventions and enhancing follow-up. Additionally, advanced nursing practice can help improve care coordination, reduce errors, and enhance patient safety. Overall, the adoption of these practices could lead to more resilient healthcare teams and better quality care.^(20)^

However, given the global nursing shortage, implementing these recommendations is difficult. Studies have reported a significant link between workload and burnout.^(21)^ Creating a safe and supportive work environment aligned with the "Magnet Hospital" model can significantly enhance nurse recruitment and retention.^(22)^ Comprehensive planning, as recommended by WHO, has demonstrated positive results for patient outcomes and working conditions.

The nursing shortage is a pressing challenge, especially in low- and middle-income countries. According to the WHO, to address this issue by 2030, an annual average increase of 8% in the number of nursing graduates is required, along with the implementation of enhanced recruitment and retention strategies. However, if current trends continue, the global nursing workforce is expected to reach only 36 million by 2030, leaving a deficit of 5.7 million nurses.^(23)^ This shortfall will predominantly affect regions such as Africa, Southeast Asia, and the Eastern Mediterranean, while countries in the Americas, Europe, and the Western Pacific will also experience significant shortages. In addition, high-income countries are attracting nurses from these regions by offering more attractive salaries and working conditions, which, while addressing their own staffing needs, create ethical concerns regarding the accessibility of healthcare. This "brain drain" from poorer countries worsens global healthcare inequalities, making it even more difficult for low-income countries to address their healthcare challenges.

In summary, high workloads in ICUs are closely linked to adverse patient outcomes, including increased mortality, more extended hospital stays, and higher costs. The pressure of excessive workloads often leads to missed care, which undermines the quality and accessibility of the care provided. To address these challenges, it is essential to establish safe staffing standards that ensure high-quality and equitable care in the ICU. Given the ongoing nursing shortage, policymakers and hospital leaders need to create environments that prioritize nurses’ well-being, support professional development, and promote sustainable workforce strategies. By doing so, they can improve both patient outcomes and the retention of a skilled nursing workforce in critical care settings.
